# Fractionated deep-inspiration breath-hold ZTE Compared with Free-breathing four-dimensional ZTE for detecting pulmonary nodules in oncological patients underwent PET/MRI

**DOI:** 10.1038/s41598-021-94702-7

**Published:** 2021-09-03

**Authors:** Chih-Yung Chang, Tse-Hao Lee, Ren-Shyan Liu, Chien-Ying Li, Bang-Hung Yang, Wen-Yi Chang, Tzu-Ping Lin, Chi-Wei Chang, Shan-Fan Yao, Tzu-Chun Wei, Chien-Yuan Lin, Charng-Chyi Shieh, Chia-Feng Lu

**Affiliations:** 1 Department of Nuclear Medicine, Taipei Veterans General Hospital, Taipei, Taiwan; 2 Department of Biomedical Imaging and Radiological Sciences, National Yang Ming Chiao Tung University, Taipei, Taiwan; 3 Division of Nuclear Medicine National Yang Ming Chiao Tung University Hospital and School of Medicine, National Yang Ming Chiao Tung University, Taipei, Taiwan; 4 School of Medicine, National Defense Medical Center, Taipei, Taiwan; 5 Department of Nuclear Medicine, Cheng-Hsin General Hospital, Taipei, Taiwan; 6 Department of Urology, Taipei Veterans General Hospital, Taipei, Taiwan; 7 Department of Urology, College of Medicine and Shu-Tien Urological Research Center, National Yang Ming Chiao Tung University, Taipei, Taiwan; 8 GE Healthcare, Taipei, Taiwan

**Keywords:** Positron-emission tomography, Magnetic resonance imaging, Cancer imaging

## Abstract

The zero echo time (ZTE) technique has improved the detection of lung nodules in PET/MRI but respiratory motion remains a challenge in lung scan. We investigated the feasibility and performance of fractionated deep-inspiration breath-hold (FDIBH) three-dimensional (3D) ZTE FDG PET/MRI for assessing lung nodules in patients with proved malignancy. Sixty patients who had undergone ZTE FDG PET/MRI and chest CT within a three-day interval were retrospectively included. Lung nodules less than 2 mm were excluded for analysis. Two physicians checked the adequacy of FDIBH ZTE and compared the lung nodule detection rates of FDIBH 3D ZTE and free-breathing (FB) four-dimensional (4D) ZTE, with chest CT as the reference standard. FDIBH resolved the effect of respiratory motion in 49 patients. The mean number and size of the pulmonary nodules identified in CT were 15 ± 31.3 per patient and 5.9 ± 4.6 mm in diameter. The overall nodule detection rate was 71% for FDIBH 3D ZTE and 70% for FB 4D ZTE (*p* = 0.73). FDIBH 3D ZTE significantly outperformed FB 4DZTE in detecting lung base nodules (72% and 68%; *p* = 0.03), especially for detecting those less than 6 mm (61% and 55%; *p* = 0.03). High inter-rater reliability for FDIBH 3D ZTE and FB 4D ZTE (k = 0.9 and 0.92) was noted. In conclusion, the capability of FDIBH 3D ZTE in respiratory motion resolution was limited with a technical failure rate of 18%. However, it could provide full expansion of the lung in a shorter scan time which enabled better detection of nodules (< 6 mm) in basal lungs, compared to FB 4D ZTE.

Lung nodules are frequently encountered in routine clinical practice^1^. For the general population, lung nodules less than 5 mm in diameter are unlikely to be malignant^2^. However, in patients with proved malignancy, it is important to detect small lung nodules, because these nodules possess the risk of lung metastases^3^. Integrated ^18^F-Fluorodeoxyglucose (FDG) PET/CT has proved its’ synergistic value in assessing these pulmonary nodules as an important part of accurate clinical staging in patients with proven malignancy^4^. On the other hand, FDG PET/MRI is an emerging modality that enables simultaneous acquisition of metabolic and morphological information with high soft-tissue contrast and reduced total radiation dose^5^. However, poor evaluation of lung nodules remains a substantial constraint to the clinical application of PET/MRI, especially for the detection of subcentimeter lung nodules.

The major limitation of pulmonary MRI is the low proton density and rapid signal decay of the lung, together with the respiratory motion of the thorax. The recent development of zero echo time (ZTE) has encouraging results for lung nodule detection and attenuation correction^6,7^. Burris et al. demonstrated the feasibility and benefit of the ultrashort echo time (UTE, minimal TE = 80 μsec) in the detection of small lung nodules ranging from 4 to 8mm^8^. Bae et al. further shortened the TE to nominal zero in their ZTE study, and provided high resolution pulmonary structural information with enhanced efficacy for detecting subcentimeter nodules using shorter scan time than UTE^7^. Zeng et al. demonstrated that the lung nodule detection rate of three-dimensional (3D) ZTE in the PET/MRI system was high even when the nodules were less than 4 mm^9^.

In addition to continued efforts to shorten the TE to enhance small nodule detection, another problem for MRI to detect lung nodule is the respiratory motion^10^. Several clinical techniques such as respiratory triggering, prospective gating, and single-breath hold have been described to deal with the respiratory motion during the MRI evaluation of lung nodules^8,11,12^. Gibiino et al. developed a free-breathing (FB) four-dimensional (4D) ZTE technique with a retrospective soft gating method for motion correction^13^. In the data acquisition, the k-space data along with physiological signals generated from respiratory bellows were collected during the whole respiratory cycle. K-space data were then weighted depending on the respiratory displacement to reconstruct the high-spatial-resolution images in different respiratory phases such as the beginning of inspiration and end-expiration. Nowadays, several clinical PET/MRI centers have suggested their protocols for the whole-body cancer survey, with additional MRI sequences specifically focusing on lung imaging. However, most of them lack the deep inspiration phase and need exceptionally long MRI acquisition times. The detection of lung base nodules may be difficult due to a lack of full expansion of the lung.

Deep-inspiration breath-hold (DIBH) has been widely used to deliver radiotherapy while limiting radiation exposure to the heart and lung^14^. It is used primarily for treating lung tumors and is subsequently applied in PET/CT imaging^15,16^. DIBH may not only allow maximal expansion of the lung but also reduce the MRI lung imaging time. This study aimed to investigate the feasibility of fractionated DIBH (FDIBH) 3D ZTE imaging for the evaluation of lung nodules in patients with proven malignancy using a hybrid PET/MRI system and to compare the nodule detecting efficacy with that of the FB 4D ZTE images.

## Methods

### Patients inclusion criteria

A survey of the picture archiving and communication system (PACS) was done, and patients with proved malignancy who underwent FDG PET/MRI and chest CT within a three-day interval from May 2018 to November 2019 were retrospectively included (n = 107). Exclusion criteria were patients who did not undergo lung ZTE (n = 19), those under the age of twenty (n = 1), and those with no lesion on the chest CT (n = 27). Sixty patients were enrolled in this study. The institutional review board of Taipei Veterans General Hospital approved this retrospective study and waived the requirement for informed consent. We confirm that all methods in our study were performed in accordance with the relevant guidelines and regulations.

### FDG PET/MRI protocol

All patients fasted for 6 h before the PET/MRI exams with their blood glucose levels checked below 160 mg/dL at the time of FDG injection. Images were acquired on a hybrid time-of-flight (TOF) PET/MRI scanner (SIGNA PET/MRI, GE Healthcare) with a 16-channel anterior coil and a 16-channel central molecular imaging array coil^9^. Sixty minutes after intravenous injection of 3 MBq/kg of FDG, the whole body PET emission scan was simultaneously acquired with the whole body non-contrast diagnostic MRI, which consisted of dual-echo gradient T1-weighted imaging and fast recovery fast spin echo T2-weighted imaging with prospective respiratory gating of the thoracic bed followed by ZTE imaging protocols. The scanned patients were allowed to breathe freely during the acquisition of the whole-body PET/MRI except for a 15-s breath-hold at end-inspiration for the thoracic bed of the dual-echo gradient T1-weighted imaging. The thoracic bed of PET emission scan was respiratory gated by the quiescent period gating (Q. Static) with offset/acquisition windows of 30/50%, respectively^9^. PET images were reconstructed using TOF ordered subset expectation maximization (OSEM) with two iterations, 28 subsets, and a Gaussian filter of 5.0 mm with a point-spread function. The respiratory gated two-point Dixon 3D volumetric inter-polated fast spoiled gradient echo sequence with free-breathing was used for attenuation correction (Q. MRAC).

### ZTE imaging protocol

ZTE lung imaging was performed consecutively with FB 4D retrospective soft gating and 3D ZTE with the FDIBH method. A respiratory bellows (GE Healthcare) was wrapped around the patient’s upper abdomen; the respiratory bellows signals were used to record the patients’ respiratory motion to retrospectively reconstruct the FB 4D ZTE image. The patients were first allowed to breathe freely for 6 min, and the k-space data were binned into three respiratory motion phases along with respiratory bellows signals collected during the whole FB cycles^12^. The end-expiratory phase images of the FB 4D ZTE were used for the detection of lung nodules. Then, the patients were asked to carefully follow the instruction of the FDIBH 3D ZTE acquisition to optimize their performance. The respiratory bellows signals were used to supervise and control the patients’ respiration during FDIBH 3D ZTE. This approach required the patients to breathe deeply and then to hold the breath uniformly and steadily for 20 s, with a two-second delay to stabilize the hold period, after which transaxial 3D ZTE acquisition was acquired^15^. The patients were directed to repeat this procedure for another three times. A single slab consists of 180 slices covered whole lung transaxial imaging was acquired in four 20-s frames to construct an FDIBH 3D ZTE image dataset. The imaging parameters for MRI acquisition were summarized in Table 1.

**Table 1:** Imaging parameters for pulmonary MRI Techniques.

Category	Dual-echo GRE T1-weighted	Fast recovery fast spin echo T2-weighted	Free-breathing four-dimensional zero echo time (FB 4D ZTE)	Fractionated deep-inspiration breath-hold zero echo time (FDIBH 3D ZTE)
Repetition time	4.7 ms	7560 ms	360 ms	556 ms
Echo time	1.9 ms	Effective TE ~ 92 ms	Nominal TE = 0	Nominal TE = 0
Acquisition type	3D, transaxial	2D, transaxial	4D, coronal	3D, transaxial
Voxel size (thickness)	1.4 mm^3^	5 mm	1.5 mm^3^	1.8 mm^3^
Flip angle	12°	111°	2°	2°
Respiratory control	Single breath-hold	Prospective respiratory gating	Retrospective soft gating	Deep-inspiration breath-hold
Approximate acquisition time	15 s	129 s	330 s	90 s

### Image analysis

Two physicians retrospectively checked the adequacy of FDIBH and read the FDG PET/MRI images blind to the chest CT information. Another senior physician read the chest CT images, and measured the longest diameter of the lung nodules on the axial chest CT images to determine the nodular size. His interpretation of the chest CT was regarded as the reference standard for the detection of lung nodules. After fusion of the Q. static PET images with FB 4D ZTE images on a GE workstation, he measured the maximum standardized uptake value (SUVmax) of all the lung nodules on these PET/ZTE images that corresponded to each reference-standard nodule using a manually adjusted cubic volume of interest (VOI). For the lung nodules visible on the reference-standard CT but not on the Q. static PET or the FB 4D ZTE, the SUVmax was not recorded. These three physicians identified every visible lung nodule in the FDG PET/MRI or chest CT images. Lung nodules less than 2 mm in diameter were excluded for analysis to minimize the effect of different slice thickness between CT (2.5 mm) and ZTE images (1.5 and 1.8 mm for FB 4D ZTE and FDIBH 3D ZTE, respectively). These nodules were classified into the right upper lobe, right middle lobe, right lower lobe, left upper lobe, and left lower lobe, according to the relative locations of these nodules to bilateral lung fissures. We defined lung base nodules as their craniocaudal locations lower than either the bifurcation of right middle lobe and right lower lobe bronchus, or the bifurcation of left lingular bronchus and left lower lobe bronchus.

### Statistical analysis

We described the patients’ baseline characteristics as means ± standard deviation and ranges for continuous variables, frequencies for categorical variables. After the pulmonary nodules were categorized by size and location of the nodules, we assessed the feasibility and efficacy of ZTE FDG PET/MRI for detecting pulmonary nodules with reference to the chest CT. The FDIBH 3D ZTE and FB 4D ZTE detection rates for pulmonary nodules were expressed as percentages, and were examined with the McNemar test using SigmaPlot (Systat Software, San Jose, CA). Inter-rater reliability of nodule detection was determined with the unadjusted Cohen’s kappa coefficient using Stata (StataCorp LP, College Station, TX). *P* < 0.05 was considered significant.

## Results

**Figure 1:**
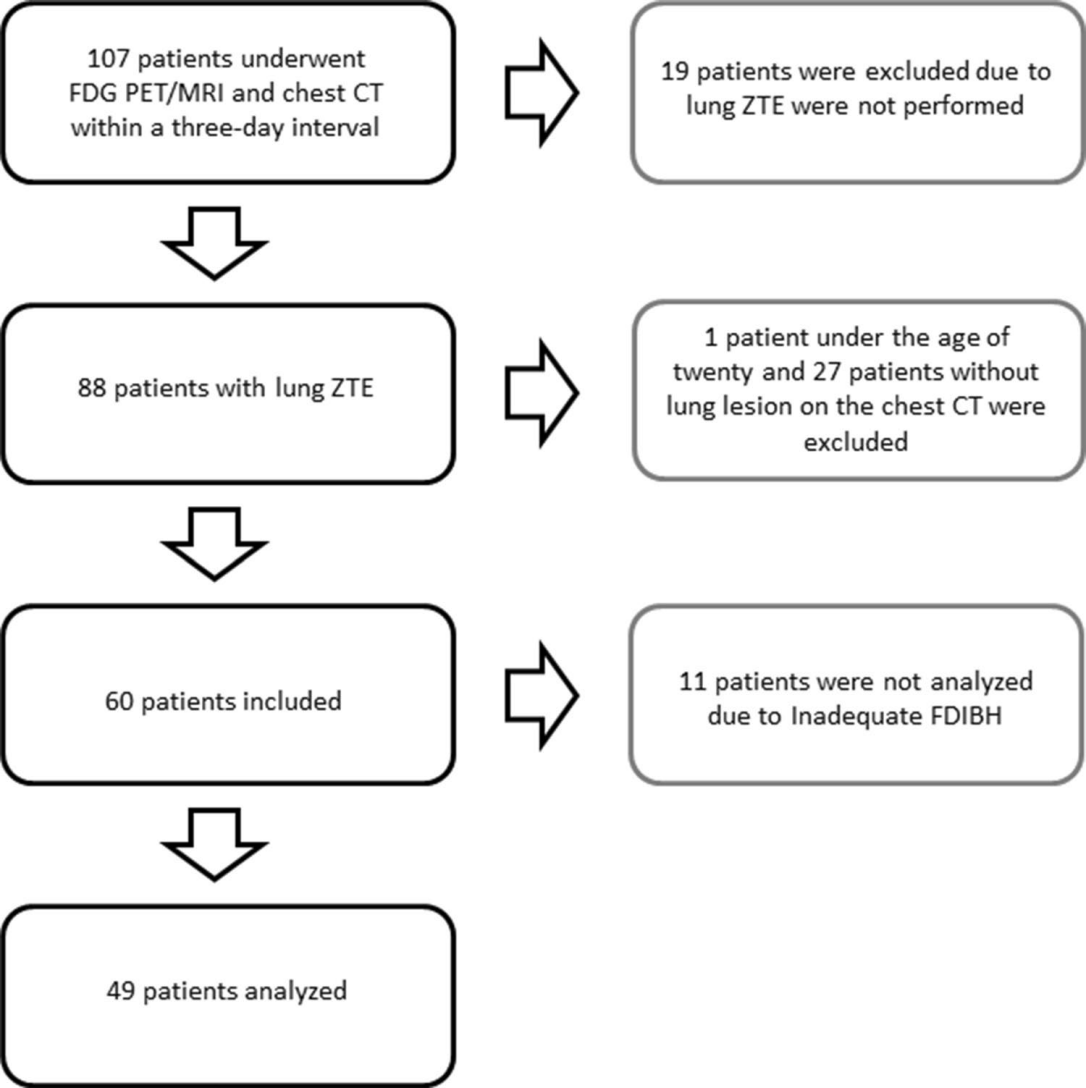
Flowchart of patient inclusion (black) and exclusion (grey).

After checking the adequacy of FDIBH, the two physicians excluded eleven patients for image analysis due to respiratory artifacts caused by inadequate FDIBH (see supplement). The rest of the 49 patients’ images were interpreted blindly to the chest CT (Fig. 1). The success rate of the FDIBH 3D ZTE scan is 82%. Table 2 summarized the patients’ information.

**Table 2:** Summary of patient information.

Total number of analyzed patients	49
Patients' age (year-old)	55.4 ± 13.8 (21–77)
Male	23 (47%)
Female	26 (53%)
Mean nodule number per patient*	15 ± 31.3 (1–160)
Mean nodule diameter at CT	5.9 ± 4.6 mm (2–28 mm)
Mean SUVmax of nodules	1.4 ± 2.7 (0.3–27)
Type of cancer**	
Lung cancer patient	20
Breast cancer	10
Esophageal cancer	6
Thyroid cancer	4
Colon cancer	2
Lymphoma	2
Oral cancer	2
Cervical cancer	1
Chondrosarcoma	1
Cholangiocarcinoma	1
Osteogenic sarcoma	1
Synovial sarcoma	1

*SUVmax* maximal standardized uptake value.

*Number of lung nodules detected by chest CT after excluding nodules less than 2 mm.

**One patient had both breast cancer and lung cancer; another patient had both thyroid cancer and lung cancer.

The mean number of pulmonary nodules identified per patient with the chest CT was 15 ± 31.3. The mean nodule diameter at CT was 5.9 mm, and 58% (423 of 733 nodules) of the nodules were less than 5 mm. The mean FDG uptake of these nodules expressed by maximal standardized uptake value (SUVmax) is 1.4 ± 2.7. There were 375 nodules classified as lung base nodules in this study. Table 3 presented nodule characteristics and distribution.

**Table 3:** Nodule characteristics.

Detectable FDG uptake	None			Positive uptake		
	635			98		

No. of nodules per size category (mm)	≧ 2 to < 4	≧ 4 to < 6	≧ 6 to < 8	≧ 8 to < 10	≧ 10 to < 15	≧ 15
	322	191	76	48	54	42

No. of nodules per lobe	Right upper	Right middle	Right lower	Left upper	Left lower	
	188	114	163	137	131	

No. of lung base nodules* per size category	≧ 2 to < 4	≧ 4 to < 6	≧ 6 to < 8	≧ 8 to < 10	≧ 10 to < 15	≧ 15
	177	79	35	31	26	27

*Nodules located lower than either the bifurcation of right middle lobe and right lower lobe bronchus, or the bifurcation of left lingular bronchus and left lower lobe bronchus.

**Figure 2:**
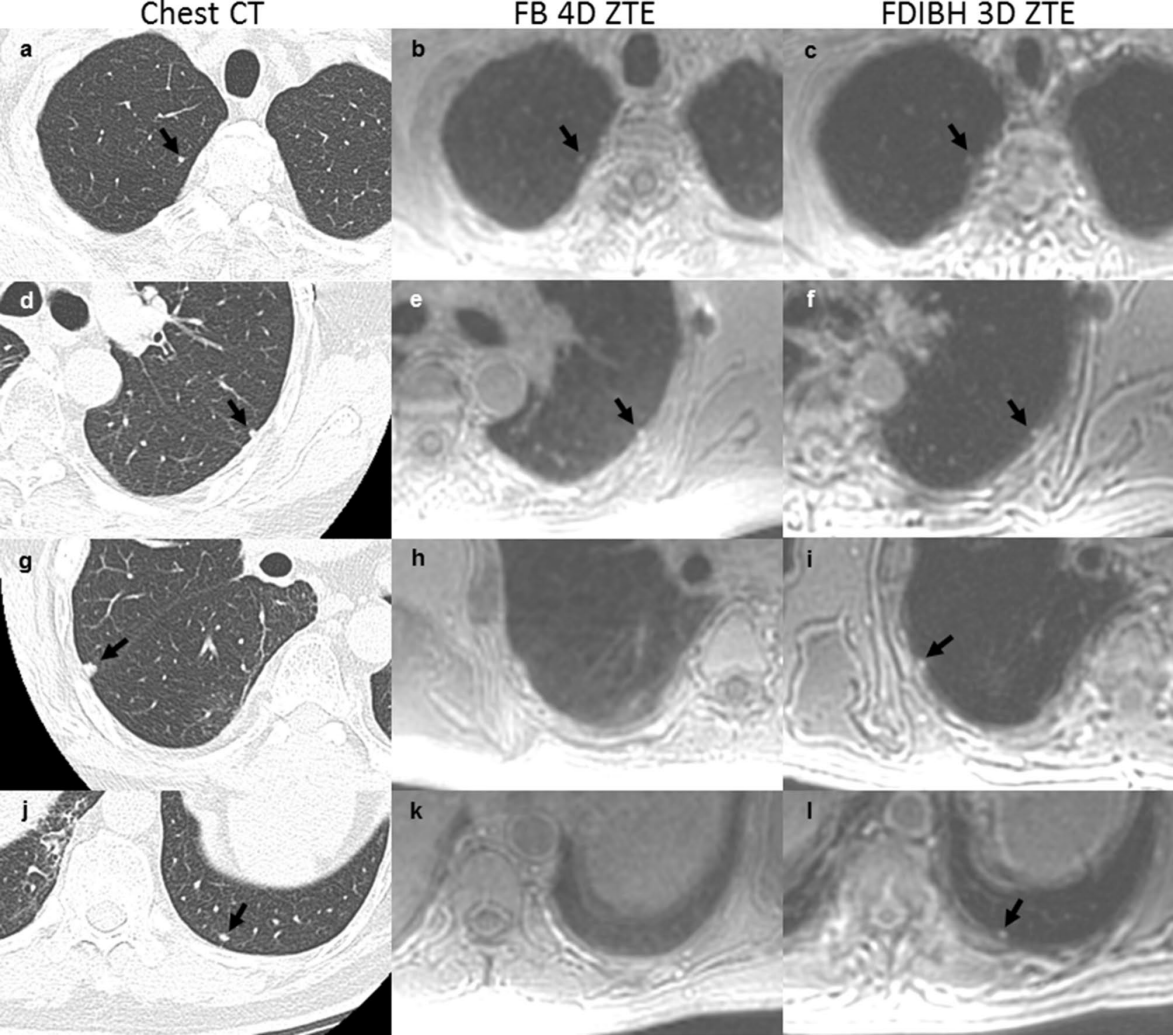
The traditional dual-echo gradient T1-weighted images and the FDG images failed to detected lung metastases in a 63-year-old male thyroid cancer patient. The FB 4D ZTE (**b**, **e**) and the FDIBH 3D ZTE (**c**, **f**) detected a 2.9 mm right upper lobe nodule and a 3 mm left upper lobe nodule. The FDIBH 3D ZTE additionally detected a 5 mm right upper lobe nodule (**i**) and another 4 mm left lower lobe nodule (**l**). All the detected nodules were identified by arrows. The lung volume of the FDIBH 3D ZTE was more similar to that of the full expansion chest CT than that of the FB 4D ZTE.

**Figure 3:**
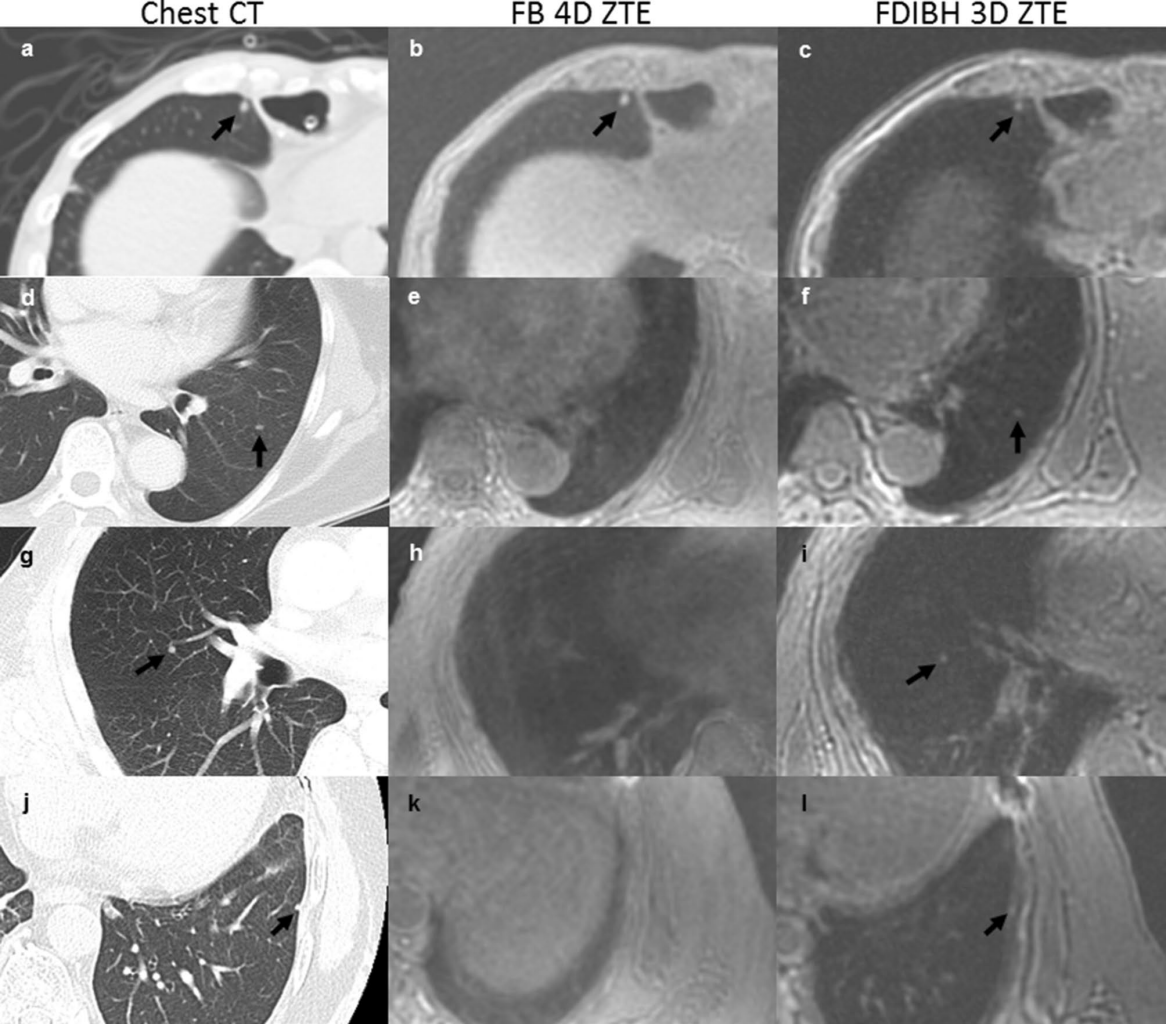
The ZTE detected lung base nodules less than 5 mm in various kinds of cancers. Both the FB 4D ZTE and the FDIBH 3D ZTE detected a 4.9 mm right middle lobe nodule (**b**, **c**) in a 50-year-old male esophageal cancer patient. The FDIBH 3D ZTE outperformed FB 4D ZTE by a 3.5 mm left lower lobe nodule (**f**) in a 64-year-old female patient with both thyroid cancer and lung cancer, a 3.3 mm right middle lobe nodule (**i**) in a 52-year-old male lung cancer patient, and a 2.3 mm left lower lobe nodule (**l**) in a 63-year-old male thyroid cancer patient. All the detected nodules were highlighted by arrows.

The overall nodule detection rate was 71% (521 of 733 nodules) for FDIBH 3D ZTE and 70% (516 of 733 nodules) for FB 4D ZTE (*p* = 0.73, Table 4). Both FDIBH3D ZTE and FB 4D ZTE had substantial detection rates (52% and 51%, *p* = 0.92, Table 4) for lung nodules at least 2 mm but smaller than 4 mm in diameter. Figure 2 demonstrated the ability of FDIBH 3D ZTE and FB 4D ZTE for detecting the small lung nodules. Furthermore, the overall detection rate of lung base nodules was significantly higher for FDIBH3D ZTE than that for FB 4D ZTE (72% and 68%, *p* = 0.03, Table 4). This benefit of FDIBH ZTE for lung base nodule primarily focused on both size categories of at least 2 mm to smaller than 4 mm (53% and 48%, *p* = 0.20, Table 4) and at least 4 mm to smaller than 6 mm (80% and 70%, *p* = 0.04, Table 4). Figure 3 demonstrated the efficacy of FDIBH 3D ZTE compared with FB 4D ZTE for the detection of small lung base nodules. The lung base nodule detection rate of FDIBH3D ZTE and FB 4D ZTE imaging for nodules 2–6 mm in diameter was 61% and 55% (*p* = 0.03). The overall inter-rater reliability for the detection of all the pulmonary nodules was high for both FDIBH 3D ZTE and FB 4D ZTE imaging (k = 0.90 and k = 0.92, respectively).

**Table 4:** All nodule and lung base nodule detection rate according to lung imaging technique.

All nodule size (mm)	≧ 2 to < 4	≧ 4 to < 6	≧ 6 to < 8	≧ 8 to < 10	≧ 10 to < 15	≧ 15	Overall
FB 4D ZTE	164/322 51%	140/191 73%	68/76 89%	48/48 100%	54/54 100%	42/42 100%	516/733 70%
FDIBH 3D ZTE	166/322 52%	145/191 76%	67/76 88%	47/48 98%	54/54 100%	42/42 100%	521/733 71%
*p*-value	0.92	0.47	1	1			0.73

Lung base nodule size (mm)	≧ 2 to < 4	≧ 4 to < 6	≧ 6 to < 8	≧ 8 to < 10	≧ 10 to < 15	≧ 15	Overall
FB 4D ZTE	85/177 48%	55/79 70%	30/35 86%	31/31 100%	26/26 100%	27/27 100%	254/375 68%
FDIBH 3D ZTE	94/177 53%	63/79 80%	30/35 86%	31/31 100%	26/26 100%	27/27 100%	271/375 72%
*p*-value	0.20	0.04*					0.03*

*FB 4D ZTE* Free-breathing four-dimensional zero echo time;

*FDIBH 3D ZTE* Fractionated deep-inspiration breath-hold three-dimensional zero echo time.

*Difference is statistically significant.

## Discussion

The built-in respiratory signal from the PET/MRI respiratory bellows can be adopted to perform FDIBH 3D ZTE lung imaging. Our preliminary result showed that FDIBH was feasible and resolved the effect of respiratory motion in 82% of the patients. FDIBH 3D ZTE PET/MRI reduced the blurring effect of respiratory motion, especially near the diaphragm, consequently improving the detection of the lung base nodules. This study demonstrated that the FDIBH 3D ZTE was both sensitive and efficient for detecting lung nodules greater than 2 mm in oncological patients who underwent FDG PET/MRI. The additional benefit for detecting lung base nodules in our FDIBH study was important because these lung base nodules had a substantial probability of representing metastatic disease in patients with proved malignancy^17^.

Notwithstanding advances in shortened TE, respiratory motion artifacts remain a major challenge for lung MRI, since adequate control of motion is not always possible in the clinical routine service^10^. The single breath-hold technique is resilient to the motion but less than ideal for ZTE PET/MRI acquisition since a few signals could be collected during a short single breath-hold and consequently, the image resolution needs to be sacrificed to achieve one breath-hold^11^. Currently, prospective gating is a commonly used technique in the 3D ZTE sequence to control motion artifacts^9^. However, this technique usually collects data within a limited end-expiration phase to allow relatively longer acquisition time and consistent breathing patterns over several FB cycles to generate a single image dataset^9^. On the other hand, retrospective soft gating techniques constantly acquire all signals during FB to reconstruct the whole image dataset with different respiratory motion states^13^. End-expiratory phase images usually demonstrate the best quality in delineating intrapulmonary structures and are used to screen lung nodules^13^. Bae et al. adopted the FB 4D ZTE techniques to obtain respiratory motion-resolved lung MRI images in all the patients regardless of patients’ lung function, with improved signal to noise ratio (SNR) and contrast to noise ratio (CNR) compared with 3D ZTE^12^. As the 4D ZTE continuously collects all the k-space data along with different respiratory phases, the artifacts due to under-sampling are reduced^12^. Furthermore, retrospective motion compensation with weighting-applied k-space data in the 4D ZTE is accomplished by weighting signals according to the deviations between actual signal and ideal target signal in each phase, resulting in enhanced signal and reduced noise of 4D ZTE images due to diminished motion within voxels^12^. However, these benefits might be partly attributed to the long acquisition time in the FB 4D ZTE, a trade-off between imaging time and imaging quality to enhance the nodule detection rate. FDIBH3D ZTE is an alternative choice with short imaging time and comparable efficacy for detecting lung nodules.

Another major concern is that these previously-reported prospective or retrospective gating techniques did not include a deep inspiratory phase with full expansion of the lung during their long tidal acquisition time. The breath-holding in deep inspiration is mandatory for ideal lung imaging for nodule detection, and has been applied to both chest CT and the PET/CT^16^. Therefore, the benefit of FDIBH 3D ZTE in detecting lung base nodules is not due to the difference in scan sequence but the difference in lung volume. Ideally, the unbiased reference standard of lung nodule detection for the end-expiration phase FB 4D ZTE should be a delicate shallow-expiratory breath-holding chest CT, or at least a CT component of a PET/CT. Furthermore, despite the advance of respiratory gating, image blurring occasionally occurred in these ZTE lung images, especially near the diaphragm and in patients with inconsistent respiratory patterns. In the present study, motion-robust ZTE lung imaging was accomplished by adequate control of respiration in FDIBH 3D ZTE in 82% of the patients. The FDIBH 3D ZTE images at deep inspiration outperformed FB 4D ZTE images for detecting lung base nodules due to the well-prepared full expansion of the lung bases. Although the 4D ZTE acquisition allows simultaneous structural motion-robust pulmonary images and functional respiratory motion information generated by the pooled volume data from different phases, the acquisition time of FB 4D ZTE was longer than that of FDIBH3D ZTE (mean, 325 s vs. 90 s), less competitive to PET/CT. Therefore, the FDIBH 3D ZTE is more efficient than the FB 4D or coronal 3D ZTE when the PET/MRI lung scan time is restricted, especially suitable for patients with normal lung function who received FDG PET/MRI for cancer staging and screening of lung metastasis without the need for pulmonary functional information.

A major strength of FB 4D ZTE is the physiological-based acquisition, which can be applied to almost every patient. Furthermore, the acquisition period of FB 4D ZTE was the same as the Q. static PET: the end-expiration period. Therefore, we could fuse these two sets of images precisely to optimize the lung nodule detection of FB 4D ZTE PET/MR. Zeng et al. reported that PET/ZTE was useful in detecting small lung nodules or nodules with a SUVmax of less than 1^9^. This benefit mainly attributed to the improved sensitivity of ZTE, since they compared the detection rate between PET/ZTE and PET/Dixon^9^. On the other hand, although breath-holding in deep inspiration was an ideal protocol for lung imaging, it was difficult to obtain both PET and ZTE MR images simultaneously with breath-holding in deep inspiration and create precisely fused images in the PET/MRI system^9^. Kawano et al. reported a solution with offline extraction and reconstruction of the list-mode dynamic PET data using the respiratory monitor recorder and the third party software^16^. They focused on lung cancers with both sizes larger than 1 cm in diameter and SUVmax above 2. They demonstrated that the breath-hold PET could enhance the FDG uptake of the lung cancers, especially for those lung cancers in the lower lung area and for the small lung cancers (> 1 cm)^16^. In our study, 58% of the nodules were less than 5 mm, and 13% of the nodules had positive FDG uptake. The benefit of combining FDIBH PET and FDIBH ZTE in comparison with the Q. Static PET/FB 4D ZTE is to be determined in a future study.

Our study had several limitations. First, this retrospective study included a small number of patients, and selection bias was inevitable. Further prospective studies with larger populations are required to corroborate our results. Second, we evaluated the nodule detection rate of FDIBH 3D ZTE and FB 4D ZTE in the same PET/MRI but not exactly the same imaging parameters with different respiratory positions. As the slice thickness was different between FDIBH 3D ZTE, FB 4D ZTE, and chest CT, we did not compare the nodular size differences between chest CT and the ZTE techniques. Another reason was that 44% of the nodules in the current study were less than 4 mm, which was prone to potential errors in size measurements. We further excluded nodules less than 2 mm for comparison in this study to ease this concern. Third, the ZTE FDG PET fusion images with ECG-gating of cardiac motion could have improved the image quality but were not discussed in this study. Lastly, while FB 4DZTE was designed for all clinical patients to improve image quality and applicability of lung ZTE imaging in PET/MRI, FDIBH 3D might not be suitable for every patient, especially for those with impaired pulmonary function and reduced capability of long breath-hold. Eleven patients in our study were excluded due to inadequate FDIBH performance.

In conclusion, we adopted the respiratory bellows signal to perform FDIBH 3D ZTE lung imaging. Our preliminary result showed that the capability of FDIBH 3D ZTE in respiratory motion resolution was limited, and resolved the respiratory motion effect in only 82% of the patients. However, this pilot study demonstrated that FDIBH 3D ZTE could be beneficial in detecting small basal lung nodules in oncology patients by providing lung images in full expansion in shorter scan time, which enabled better detection of nodules (< 6 mm) in basal lungs, compared to FB 4D ZTE.

## Supplementary Information

Supplementary Information.
