# Supplementary material for: Fractionated deep-inspiration breath-hold ZTE Compared with Free-breathing four-dimensional ZTE for detecting pulmonary nodules in oncological patients underwent PET/MRI
Source: Sci Rep. 2021 Sep 3;11:17636. doi: 10.1038/s41598-021-94702-7 (PMC8417270; doi:10.1038/s41598-021-94702-7)
Supplement: Supplementary file 1 — Supplementary Information. [file 41598_2021_94702_MOESM1_ESM.docx]

**Fractionated Deep-inspiration breath-hold ZTE Compared with Free-breathing four-dimensional ZTE for Detecting Pulmonary Nodules in Oncological Patients underwent PET/MRI**

Chih-Yung Chang^1,2,3,4^, Tse-Hao Lee^1^, Ren-Shyan Liu^2,5^, Chien-Ying Li^1,2^, Bang-Hung Yang^1,2^, Wen-Yi Chang^1,2^, Tzu-Ping Lin^6,7^*, Chi-Wei Chang^1,2^, Shan-Fan Yao^1^, Tzu-Chun Wei^6,7^, Chien-Yuan Lin^8^, Charng-Chyi Shieh^8^, Chia-Feng Lu^2^*

^1^Department of Nuclear Medicine, Taipei Veterans General Hospital, Taipei, Taiwan

^2^Department of Biomedical Imaging and Radiological Sciences, National Yang Ming Chiao Tung University, Taipei, Taiwan

^3^Division of Nuclear Medicine National Yang Ming Chiao Tung University Hospital and School of Medicine, National Yang Ming Chiao Tung University, Taipei, Taiwan

^4^School of Medicine, National Defense Medical Center, Taipei, Taiwan

^5^Department of Nuclear Medicine, Cheng-Hsin General Hospital, Taipei, Taiwan

^6^Department of Urology, Taipei Veterans General Hospital, Taipei, Taiwan

^7^Department of Urology, College of Medicine and Shu-Tien Urological Research Center, National Yang Ming Chiao Tung University, Taipei, Taiwan

^8^GE Healthcare, Taipei, Taiwan

*Corresponding author:

*Chia-Feng Lu, PhD

Department of Biomedical Imaging and Radiological Sciences, National Yang Ming Chiao Tung University, No. 155, Sec. 2, Linong Street, Beitou District, Taipei City 112, Taiwan

Tel: +886-2-28267308

Fax: +886-2-28201092

Email: [alvin4016@nycu.edu.tw](mailto:alvin4016@nycu.edu.tw)

*Tzu-Ping Lin, MD, PhD

Department of Urology, Taipei Veterans General Hospital, No. 201, Sec. 2, Shipai Road, Beitou District, Taipei City 112, Taiwan

Tel: +886-2-28757519

Fax: +886-2-28757540

Email: tplin63@gmail.com

Chia-Feng Lu and Tzu-Ping Lin contributed equally.

**Supplemental Material**


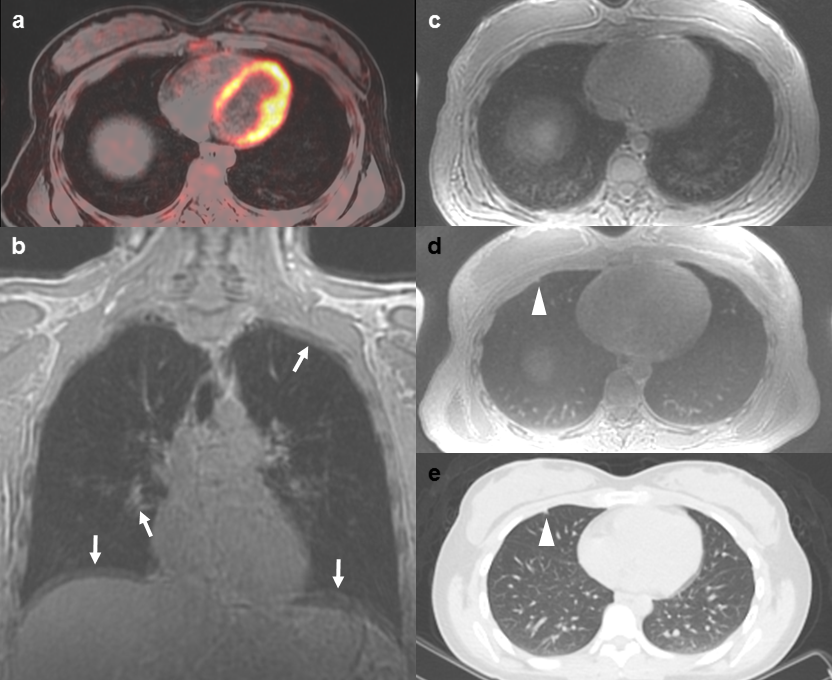


Illustration of quality check for the adequacy of FDIBH 3D ZTE during FDG PET/MR imaging (a) was demonstrated. FDIBH 3D coronal ZTE images (b) with misalignment of the diaphragm (arrow), the motion of chest wall (arrow), or blurring in the lung parenchyma (arrow) were excluded for analysis. The inadequate FDIBH 3D ZTE with blurring, ghosting, signal dropouts, and undesirable signal enhancement led to no detection of a 3.5 mm right middle lobe lung nodule (c), which was evident on the corresponding FB 4D ZTE (d, arrowhead) and chest CT (e, arrowhead).
